# Value of multiplex PCR for detection of antimicrobial resistance in samples retrieved from patients with orthopaedic infections

**DOI:** 10.1186/s12866-020-01741-7

**Published:** 2020-04-14

**Authors:** Irene Katharina Sigmund, Nora Renz, Susanne Feihl, Christian Morgenstern, Sabrina Cabric, Andrej Trampuz

**Affiliations:** 1 Charité – Universitätsmedizin Berlin, corporate member of Freie Universität Berlin, Humboldt-Universität zu Berlin, and Berlin Institute of Health, Center for Musculoskeletal Surgery (CMSC), Charitéplatz 1, 10117 Berlin, Germany; 2 Department of Orthopaedics and Trauma Surgery, Medical University of Vienna, Spitalgasse 23, 1090 Vienna, Austria; 3 Berlin Institute of Health Center for Regenerative Therapies (BCRT), Föhrer Strasse 15, 13353 Berlin, Germany

**Keywords:** Multiplex PCR, Periprosthetic joint infection, Antimicrobial resistance, Sonication, Molecular methods, Diagnosis

## Abstract

**Background:**

The performance of multiplex PCR (mPCR) for detection of antimicrobial resistance from clinical isolates is unknown. We assessed the ability of mPCR to analyse resistance genes directly from clinical samples.

Patients with orthopedic infections were prospectively included. Phenotypical and genotypical resistance was evaluated in clinical samples (synovial and sonication fluid) where identical pathogens were identified by culture and mPCR.

**Result:**

A total of 94 samples were analysed, including 60 sonication fluid and 34 synovial fluid samples. For coagulase-negative staphylococcus strains, mPCR detected resistance to oxacillin in 10 of 23 isolates (44%) and to rifampin in none of 6 isolates. For *S. aureus* isolates, detection rate of oxacillin and rifampin-resistance was 100% (2/2 and 1/1, respectively). Fluoroquinolone-resistance was confirmed by mPCR in all 3 isolates of Enterobacteriaceae, in enterococci resistance to aminoglycoside-high level was detected in 1 of 3 isolates (33%) and in streptococci resistance to macrolides/lincosamides in none of 2 isolates. The overall sensitivity for different pathogens and antimicrobials was 46% and specificity 95%, the median concordance was 80% (range, 57–100%). Full agreement was observed for oxacillin in *S. aureus*, vancomycin in enterococci, carbapenems/cephalosporins in Enterobacteriaceae and rifampin in *Cutibacterium* species.

**Conclusion:**

The overall sensitivity for detection of antimicrobial resistance by mPCR directly from clinical samples was low. False-negative mPCR results occurred mainly in coagulase-negative staphylococci, especially for oxacillin and rifampin. However, the specificity of mPCR was high and a positive result reliably predicted antimicrobial resistance. Including universal primers in the PCR test assay may improve the detection rate but requires additional sequencing step.

**Trial registration:**

www.clinicaltrials.gov No. NCT02530229, registered at 21 August 2015 (retrospectively registered).

## Background

An interdisciplinary approach including surgical and antimicrobial treatment is crucial to eradicate bone and joint infection, including periprosthetic joint infections (PJI) and infections after internal bone fixation [1]. Treatment strategies are guided by the type of causative pathogen and its antimicrobial susceptibility, with special focus on biofilm-active antibiotics [2].

Previous studies demonstrated limited sensitivity of periprosthetic tissue culture (45–71%) and synovial fluid culture (52–64%) for microbial detection [3–8]. Cultures remain negative in particular in low- burden, chronic low-grade infections or in case of preceding antimicrobial therapy [9, 10]. Moreover, phenotypical antimicrobial testing using conventional cultures requires several days in mixed infections and if slow-growing microorganisms are involved*.* Therefore, new techniques were developed to improve the diagnostic yield, such as sonication of retrieved implants and broad-range PCR of sonication fluid, improving sensitivities to 73–89% [3, 4, 10, 11]. In a further study, inoculation of sonication fluid into blood culture bottles further improved the diagnosis of orthopaedic implant-associated infections and reduced the time to culture positivity [12].

The value of multiplex PCR (mPCR) in synovial fluid, periprosthetic tissue and sonication fluid has been recently extensively investigated [6, 13–18]. Advantages of the mPCR technique are rapid identification of the causative pathogen and potentially detection of genetic markers for clinically relevant antimicrobial resistances [18]. However, limited data exists on the performance of mPCR for the detection of antimicrobial resistance markers in the clinical setting.

In this study, we assessed the value of a commercial mPCR assay (Unyvero i60 ITI) in genotypical detection of antimicrobial resistance, considering conventional culture the gold standard. In addition, we evaluated the concordance of susceptibility test results deriving from mPCR and culture in pathogens isolated from synovial and sonication fluid.

## Results

### Patient demographics

Ninety-four samples of 82 patients with a median age of 75 years (range, 28–98 years) were analysed, 47 patients (57%) were female. The 60 sonication fluid samples originated from 38 infected prostheses (20 knee, 15 hip, 2 shoulder, and 1 elbow prosthesis) and 22 osteosyntheses (localized in the spine in 9 patients, tibia in 6, ankle in 2, femur in 2, humerus in 1, clavicle in 1, and elbow in 1 patient). The 34 synovial fluids were harvested from 31 infected prosthetic (13 knees and 18 hips) and 3 native septic joints (two knee and one hip joint).

### Microbiological characteristics

The detected pathogens in sonication and synovial fluid samples are summarized in Table 1. The most frequently isolated pathogens were *Staphylococcus* spp. (52%), followed by Enterobacteriaceae (15%) and *Streptococcus agalactiae* (12%). Eighty-five monomicrobial and 9 polymicrobial infections were observed. In 12 patients, both sonication and synovial fluid were analysed and yielded the identical pathogen.

**Table 1:** Distribution of detected pathogens

Microbiology	Sonication fluid (***n*** = 60)	Synovial fluid (***n*** = 34)	Total (***n*** = 94)
Coagulase-negative staphylococci	21	12	33 (35%)
*Staphylococcus aureus*	9	7	16 (17%)
*Streptococcus agalactiae*	4	7	11 (12%)
*Enterococcus* spp.^a^	8	2	10 (11%)
*Escherichia coli*	4	5	9 (10%)
*Cutibacterium* spp.^b^	8	1	9 (10%)
*Enterobacter cloacae*	3	0	3 (3%)
*Finegoldia magna*	1	0	1 (1%)
*Proteus* spp*.*	1	0	1 (1%)
*Klebsiella pneumoniae*	1	0	1 (1%)

^a^Including 9 isolates of *E. faecalis* and 1 isolate of *E. faecium*

^b^Including 8 isolates of *C. acnes* and 1 isolate of *C. avidum*

### Performance of resistance gene detection and its concordance with phenotypical resistance testing

The detection rate of vancomycin resistance by mPCR in *Enterococcus* spp. could not be assessed as no resistance was detected by conventional culture. The same applies for Enterobacteriaceae and aminoglycosides, carbapenems, third-generation cephalosporins, as well as for *Cutibacterium* spp. and rifampin.

Performance of mPCR and concordance of phenotypical and genotypical susceptibility testing is summarized in Table 2 (Additional file 1). Overall, a median sensitivity of 46% and specificity of 95% was shown, with a median concordance of 80%. There was no difference seen regarding number of discordant pairs comparing synovial and sonication fluid.

**Table 2:** Performance of mPCR and concordance of phenotypical and genotypical antimicrobial susceptibility of all tested isolates

Pathogen/Antimicrobial	Gene target	Susceptible by mPCR [no.]	Resistant by mPCR [no.]	Susceptible by culture [no.]	Resistant by culture [no.]	Sensitivity [%] (95% CI)	Specificity [%] (95% CI)	Concordance [%] (95% CI)	NPV [%] (95% CI)	PPV [%] (95% CI)
Coagulase-negative staphylococci										
Oxacillin (*n* = 30)	*mecA, mecC*	20	10	7	23	44 (26–63)	100 (59–100)	57 (39–74)	35 (14–56)	100 (100)
Aminoglycosides (*n* = 32)	*aac(6′)/aph(2″)*	23	9	13	19	42 (23–64)	92 (64–100)	63 (46–79)	52 (32–73)	89 (68–100)
Macrolides/lincos-amides (*n* = 33)	*ermA, ermC*	19	14	14	19	58 (36–77)	79 (51–93)	67 (51–83)	58 (36–80)	79 (57–100)
Rifampin (*n* = 28)	*rpoB*	28	0	22	6	0 (0–46)	100 (85–100)	78 (59–91)	79 (79)	–
*Staphylococcus aureus*										
Oxacillin (*n* = 16)	*mecA, mecC*	14	2	14	2	100 (29–100)	100 (74–100)	100 (100)	100 (100)	100 (100)
Aminoglycosides (n = 16)	*aac(6′)/aph(2″)*	16	0	15	1	0 (0–98)	100 (78–100)	–	94 (94)	94 (70–100)
Macrolides/lincos-amides (*n* = 16)	*ermA, ermC*	14	2	12	4	50 (15–85)	100 (71–100)	88 (71–100)	86 (67–100)	100 (100)
Rifampin (*n* = 15)	*rpoB*	13	2	14	1	100 (17–100)	93 (66–100)	93 (81–100)	100 (100)	50 (0–100)
*Enterococcus* spp.										
Aminoglycosides (high-level) (*n* = 7)	*aac(6′)/aph(2″)*	6	1	4	3	33 (6–80)	100 (45–100)	71 (38–100)	67 (29–100)	100 (100)
Vancomycin (*n* = 10)	*vanA, vanB*	10	0	10	0	–	100 (69–100)	100 (69–100)	100 (100)	–
Enterobacteriaceae										
Aminoglycosides (*n* = 14)	*aacA4*	10	4	14	0	–	71 (42–92)	71 (42–92)	100	0
Carbapenems (*n* = 14)	*bla*_*vim*_*, bla*_*imp*_*, bla*_*kpc*_*, bla*_*ndm*_*, bla*_*oxa-23*_*, bla*_*oxa-24*_*, bla*_*oxa-48*_*, bla*_*oxa-58*_	14	0	14	0	–	100 (77–100)	100 (77–100)	100	–
Third-generation cephalosporins (*n* = 14)	*ctx-M*	14	0	14	0	–	100 (77–100)	100 (77–100)	100	–
Fluoroquinolones^a^ (*n* = 9)	*gyrA83, gyrA87*	5	4	6	3	100 (38–100)	83 (42–98)	89 (68–100)	100 (100)	75 (33-100)
*Streptococcus agalactiae*										
Macrolides/lincosamides (*n* = 10)	*ermA, ermC*	10	0	8	2	0 (0–84)	100 (63–100)	80 (44–97)	80 (80)	–
*Cutibacterium spp*										
Rifampin (*n* = 6)	*rpoB*	6	0	6	0	–	100 (54–100)	100 (54–100)	100 (100)	–
Total, median (range)						46 (36–56)	95 (90–97)	80 (75–84)	80 (74–85)	79 (68–91)

Note: Sensitivity and specificity of mPCR considering culture testing (VITEK) as gold standard

^a^Only *E. coli* isolates were tested for fluoroquinolone resistance by mPCR

#### *Staphylococcus* spp

Among *Staphylococcus* spp. isolates, concordance of phenotypical and genotypical susceptibility to oxacillin, aminoglycosides, macrolides/lincosamides and rifampin was 72, 73, 73 and 84%, respectively. The mPCR detected oxacillin resistance genes in 10 of 23 samples associated with oxacillin-resistant coagulase-negative staphylococci positive cultures (44%), whereas both oxacillin-resistant *S. aureus* strains detected by culture were found to be resistant by mPCR (sensitivity 100%). In coagulase-negative staphylococci, none of the rifampin resistance determined by culture was detected by mPCR; in *S. aureus* isolates, one true positive and one false negative rifampin resistance was detected by mPCR, resulting in a NPV of 100% and a PPV of 50%.

#### *Enterococcus* spp

The vancomycin antimicrobial resistance testing showed 100% agreement in 10 isolates (all tested susceptible). Among 7 tested strains, one isolate was tested resistant to aminoglycosides with both methods. Genotypical high-level resistance to aminoglycosides was determined in 1 sample, whereas phenotypical resistance was determined by culture in 3 isolates. Thus, mPCR missed the resistance to aminoglycosides detected through *aac(6′)*/*aph(2″)* in 2 samples (66%). The resulting concordance was 71% with a sensitivity, specificity, NPV and PPV of 33, 100, 67 and 100%, respectively.

#### Enterobacteriaceae

The overall agreement for the testing of different antimicrobial agents in 14 Enterobacteriaceae isolates was good with mPCR test results showing NPV of 100% for all antimicrobial agents. mPCR detected resistance to fluoroquinolones in all 3 isolates. False-positive resistance was detected for aminoglycosides in 4 patients, resulting in a PPV of 0% and for fluoroquinolones in 1 patient with a PPV of 75%.

#### *Streptococcus* spp

Of 10 *Streptococcus agalactiae* isolates, lincosamides by culture/lincosamides by culture and ten by mPCR, resulting in a concordance and NPV of 80% and a specificity of 100%. As no resistant strain was detected, PPV could not be assessed.

#### Anaerobes

For rifampin, all 6 isolates of *Cutibacterium* spp. showed concordance (susceptibility by culture and mPCR). In one sonication fluid sample, *Finegoldia magna* was identified by culture and mPCR. This pathogen was susceptible to amoxicillin/clavulanic acid, penicillin, piperacillin/tazobactam, imipenem, clindamycin, rifampin and levofloxacin by conventional susceptibility testing using culture. However, no markers are available for resistance testing for this pathogen using the mPCR system.

## Discussion

Rapid identification of a pathogen and its susceptibility pattern allows effective optimization of the antimicrobial treatment and prevention of emergence of resistance. While conventional culture is the gold-standard microbiological method, molecular methods are increasingly used in the clinical routine. However, limitations of molecular detection of drug-resistance genes need to be considered, in particular the fact that genotypic drug-resistance identification does not conclusively corresponds to the phenotype [19]. In addition, focusing on the detection of a certain selection of genes with mPCR, the diversity of all bacterial resistance genes is not represented.

The sensitivity and specificity of mPCR for pathogen detection directly from clinical samples found in the published sub-cohorts ranged in sonication fluid from 51 to 71% and 92 to 94%, respectively, and in synovial fluid from 23 to 60% and 89 to 91%, respectively [6–8, 17]. Previous publications using mPCR for pathogen identification reported similar sensitivities ranging from 51 to 96% and specificities from 94 to 100% [13, 16, 18, 20, 21]. However, limited data exists on the performance of the mPCR system for detection of resistance genes and their agreement on conventional antimicrobial susceptibility testing in patients with bone and joint infections [18, 22].

A recent study [23] employing the first generation of the multiplex PCR device reported a low detection rate (6%) of *mecA* and *mecC* genes in methicillin-resistant staphylococci (not specified which subspecies), which increased to 35% when using the second generation of the mPCR cartridge. In our cohort, we observed a detection rate of 48% (12 of 25 isolates) for oxacillin resistance. However, the oxacillin-resistance detection rate in *S. aureus* was considerably higher than in coagulase-negative staphylococci (100% versus 44%). The limited detection of oxacillin resistance in coagulase-negative staphylococci by mPCR may be explained by the low microbial burden usually seen in low grade infections caused by less virulent staphylococci, which probably does not reach the detection limit of the mPCR system of *mecA* and *mecC*, estimated at ~ 10^4^ and ~ 10^6^ DNA copies/ml, respectively. This hypothesis is corroborated by the fact, that the concordance in infections caused by *S. aureus* – usually acute infections - was considerably higher. Furthermore, a great diversity of the staphylococcal cassette chromosome *mec* in coagulase-negative staphylococci may contribute to a reduced detection rate of oxacillin resistance [24].

From the clinical viewpoint, susceptibility to rifampin is of paramount relevance in implant-associated infections caused by staphylococci and *Cutibacterium* spp., as this antibiotic is the only biofilm-active antibiotic for these microorganisms [25, 26]. Pre-operative knowledge of rifampin resistance influences the surgical decision, as a lack of biofilm-active treatment usually discourages retention of the implant in acute infections and a one-stage exchange in chronic infections [1]. In our cohort, the concordance between genotypical and phenotypical evaluation for rifampin was moderate for *Staphylococcus* spp. (84%) with 86% false-negative test-result (i.e. phenotypically resistant and genotypically susceptible) and excellent for *Cutibacterium* spp. (100%). However, the cartridge is not validated for rifampin susceptibility testing in *Cutibacterium* spp. and coagulase-negative staphylococci. When analysing *S. aureus* only, for which the system is validated, a concordance of 93% with a negative predictive value of 100% was obtained. Similarly, fluoroquinolones represent the biofilm-active agent for implant-associated infections caused by gram-negative bacteria [27]. For all analysed *E. coli* strains, a concordance of 89% for fluoroquinolones with a 100% NPV was detected.

With 71 to 73%, the concordance of phenotypical and genotypical testing for aminoglycosides was in the lower range for all tested pathogens, i.e. staphylococci, enterococci and gram-negative rods. For enterococci and streptococci, the NPV was low due to missed resistance by mPCR. In contrast, in gram-negative rods false-positive mPCR test resulted in low PPV (0%).

We acknowledge several limitations of this study. First, given the low number of rifampin-resistant staphylococci and fluoroquinolone-resistant Enterobacteriaceae, its value for detection of pathogens causing difficult-to-treat infections is limited and their predictive values from our study should be taken with caution. Additionally, we could not assess detection rates for several resistance genes, as we did not include any isolates tested resistant by conventional culture (e.g. gram-negative bacilli resistant to aminoglycosides, third generation cephalosporines and carbapenems). Another limitation represents the inclusion of exclusively those clinical samples, which were concordant in pathogen detection by culture and mPCR. The performance of mPCR in discordant samples is unknown and the specificity results of this study may be overestimated. Furthermore, we have not investigated the performance of mPCR in viable but non culturable (VBNC) condition, where the number of DNA copies may be much lower than that in growing condition. Finally, an analysis between the quantitative MIC values (i.e., strength of resistance) and detectability of resistance genes (i.e., numbers of DNA copies) was not performed in our study, which might provide additional insights in the performance of mPCR for detection of antimicrobial resistance.

## Conclusion

The overall sensitivity for detection of antimicrobial resistance by mPCR directly from clinical samples was low. Due to low sensitivity the evaluated commercial mPCR system cannot replace conventional antimicrobial resistance testing. False-negative mPCR results occurred mainly in coagulase-negative staphylococci, especially for oxacillin and rifampin. However, the specificity of mPCR was high and a positive result reliably predicts resistance and for some pathogens and antibiotics full agreement was observed, including for oxacillin in *S. aureus*, vancomycin in enterococci, carbapenems/cephalosporins in Enterobacteriaceae and rifampin in *Cutibacterium* species. Including universal primers in the PCR test assay may improve the detection rate but requires additional sequencing step.

## Methods

### Study design

**Fig. 1:**
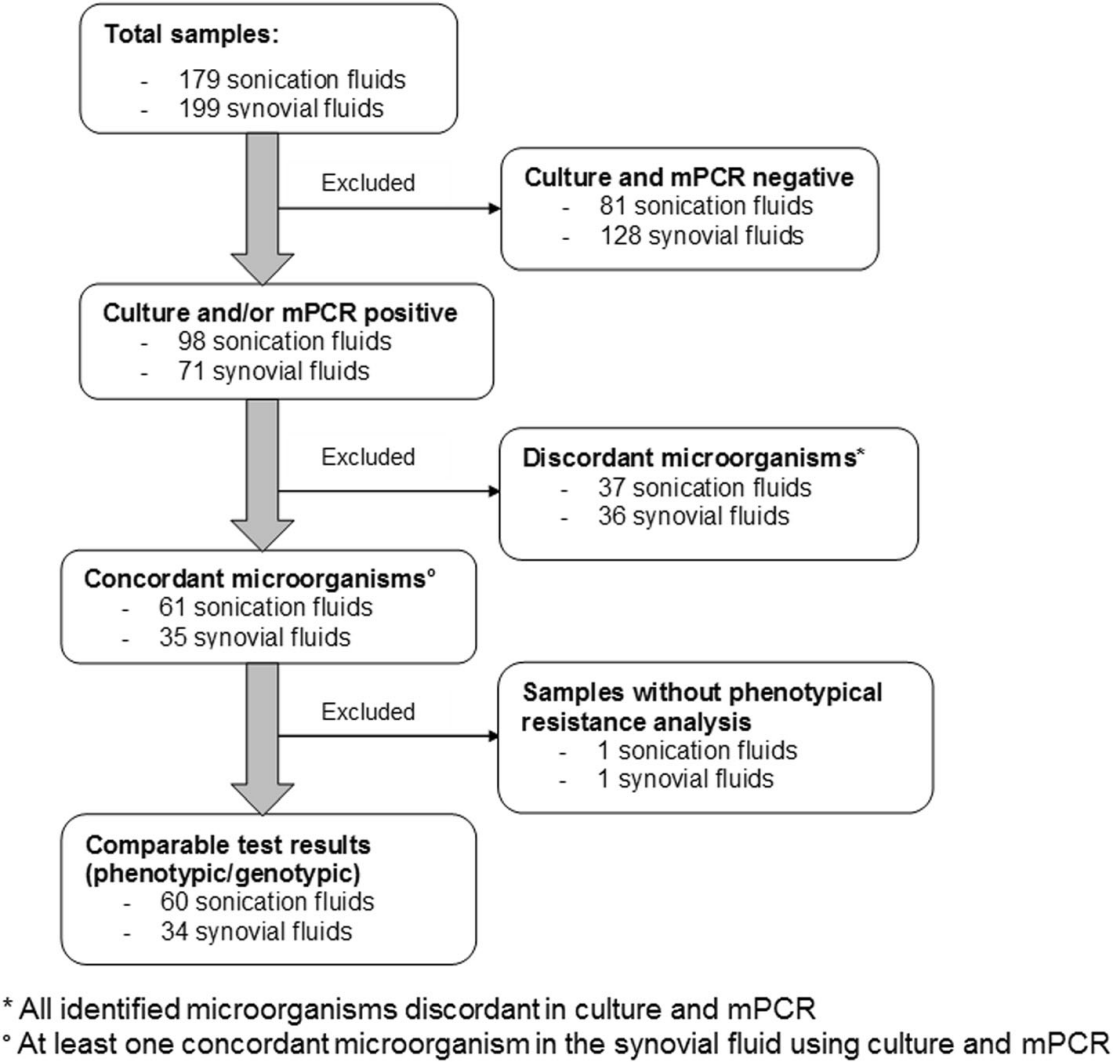
Study selection flow diagram

This prospective single-center study (public clinical trial identification at www.clinicaltrials.gov NCT02530229) was conducted in accordance with the Declaration of Helsinki. Approval of the institutional review board was obtained (EA1/306/14) and patients provided written informed consent before study inclusion. The results of mPCR were not used to guide antimicrobial therapy. Data analysed in this study represent a subpopulation of previously published cohorts analysing the performance of mPCR with regards to detection of bacteria in synovial fluid of native joints [7] and periprosthetic joints [17], as well as in sonication fluid from removed joint prostheses [6] and other orthopaedic hardware [8]. Among 378 samples (179 sonication fluids [128 prostheses, 51 osteosyntheses], 199 synovial fluids [142 prosthetic joints, 57 native joints]) enrolled in the above-mentioned published cohort studies, 94 samples detected the same microorganisms using mPCR and conventional culture. Two-hundred-nine specimens with no microbial growth, 73 patients with discordant microorganisms and two cases, where no phenotypical drug resistance analysis was performed, were excluded. A study selection flow diagram is shown in Fig. 1.

### Study population

Consecutive adult patients who had undergone diagnostic joint aspiration for suspected septic arthritis or revision surgery for implant-associated bone infection between November 2014 and October 2015 were included. Four patients in whom sonication fluid of retrieved osteosynthesis was investigated, were receiving antimicrobial treatment at time of explantation. No other patients had been pre-treated with antibiotics.

### Joint aspiration

Synovial fluid was aspirated by an orthopaedic surgeon according to standardized aseptic technique. After skin preparation, synovial fluid was collected using a sterile 18-gauge spinal needle and a 10-ml syringe. If no synovial fluid was obtained, the needle was repositioned without withdrawing it through the skin.

### Synovial fluid cultures

0.1 milliliter of synovial fluid was inoculated onto aerobic and anaerobic plates (sheep blood agar, chocolate agar, and Schädler anaerobic agar), and 1 ml was inoculated in thioglycolate broth. Agar plates were incubated at 37 °C under aerobic and anaerobic conditions for 14 days and inspected daily for microbial growth. Microbial identification was performed using Vitek2 (bioMérieux, Nürtingen, Germany) or a matrix-associated laser desorption/ionization-time of flight mass spectrometer (bioMérieux). The antimicrobial susceptibility testing was conducted using Vitek2 and the minimum inhibitory concentration (MIC) values were determined and interpreted according to EUCAST standards. For mPCR analysis, 2–5 ml of the synovial or 10–50 ml of sonication fluid was transferred to a sterile vial and stored at − 80 °C until processing.

### Removal and transport of orthopedic hardware

The joint prosthesis components (metal, ceramic or polyethylene parts) or other fracture fixation devices (plates, nails or screws) were removed in the operating room. After removal, the hardware components were transported to the microbiological laboratory in a sterile air-tight container (Lock&Lock, Frankfurt am Main, Germany) and processed within 6 hours.

### Sonication procedure

In the microbiology laboratory, normal saline was added to the container, covering at least 80% of the hardware. The container was vortexed for 30 s and sonicated for 1 minute at 40 kHz and 0.2 W/cm^2^ (BactoSonic, Bandelin electronic, Berlin, Germany). Additional vortexing was conducted for 30 s before the sonication fluid was plated. Then, 0.1 ml of the sonication fluid was inoculated onto agar plates and 1 ml was inoculated in thioglycolate broth. Microorganisms were enumerated (i.e. number of colony-forming unit [CFU]/ml sonication fluid). Microbial identification and antimicrobial susceptibility testing were performed as described above for synovial fluid.

### Multiplex PCR analysis

The Unyvero i60 ITI application (Unyvero i60, Curetis, Holzgerlingen, Germany, second generation), designed for a semi-quantitative DNA determination parallelly performing eight multiplex PCR reactions, was used. This instrument is capable to detect 114 nucleic acids and 20 drug resistance markers. Fifty milliliter of the sonication fluid were first centrifuged at 4000 x g at 4 °C for 20 min (Microcentrifuge 5427R, Eppendorf, Wesseling-Berzdorf, Germany). The supernatant was discarded and 180 μl of the pellet were further processed. The synovial fluid was processed directly without centrifugation. The Unyvero i60 ITI application was used according to the manufacturer’s protocol. In brief, the pre-treated sample was treated with proteinase K for 30 min, master mix was added and inserted into the Unyvero ITI Cartridge. The cartridge device is equipped with eight separate chambers, a corresponding number of arrays, reagent containers and a DNA purification column. The assembled and closed Unyvero ITI Cartridge was inserted into the Unyvero Analyzer, which then automatically processed the sample within 5 hours. The microbial identification and antibiotic resistance markers are displayed on the screen of the instrument. A sample was considered positive if at least one of the analytes (pathogens) reached the threshold of 10^4^ DNA copies/ml.

According to the manufacturer, the resistance markers *aacA4*, *ctx-M*, *ermA*, *gyrA83*, *mecA*, *vanA* and the beta-lactamase genes *bla*_*ndm*_*, bla*_*oxa-23*_*, bla*_*oxa-48*_*, bla*_*oxa-58*_ were detected at a concentration of 10^4^ DNA copies/ml; the resistance markers *aac(6′)/aph(2″), gyrA87, bla*_*imp*_*, bla*_*kpc*_*, bla*_*oxa-24*_, and *bla*_*vim*_ were detected at a concentration of 10^5^ DNA copies/ml; and the resistance markers *ermC, mecC, rpoB*, and *vanB* at a concentration of 10^6^ DNA copies/ml. The range of antibiotic resistance genes detected with the mPCR system is shown in Table 3.

**Table 3:** Resistance markers detected by the multiplex PCR test panel

Antibiotics	Resistance markers
Oxacillin	*mecA, mecC*
Aminoglycosides	*aac(6′)/aph(2″), aacA4*
Macrolides/lincosamides	*ermA, ermC*
Vancomycin	*vanA, vanB*
Rifampin^a^	*rpoB*
Third-generation cephalosporins	*ctx-M*
Carbapenems	*bla*_*vim*_*, bla*_*imp*_*, bla*_*kpc*_*, bla*_*ndm*_*, bla*_*oxa-23*_*, bla*_*oxa-24*_*, bla*_*oxa-48*_*, bla*_*oxa-58*_
Fluoroquinolones^b^	*gyrA83, gyrA87*

^a^Rifampin resistance testing is only validated for *Staphylococcus aureus*

^b^Fluoroquinolone resistance testing is only validated for *Escherichia coli* among gram-negative bacteria

### Statistical analysis

Categorical variables are described as absolute frequencies and percentages. Metrical variables are expressed by median and range, as appropriate. The performance of mPCR (i.e. sensitivity and specificity) and its positive predictive value (PPV) and negative predictive value (NPV) were assessed in terms of detection of resistance, using phenotypical testing by conventional culture as gold standard. Concordance analysis was performed comparing phenotypical and genotypical resistance test results, for substances, for which genotypical resistance markers were included in the mPCR cartridge. The software packages XLSTATPM (version 2017; XLSTAT; Addinsoft, New York, NY, USA).

## Supplementary information

**Additional file 1.** Raw data of the concordant microorganisms and their detected resistances by mPCR and culture.

## Data Availability

All data generated or analysed during this study are included in this published article.

## Abbreviations

CFU: Colony-forming unit
DNA: Deoxyribonucleic acid
MIC: Minimum inhibitory concentration
mPCR: Multiplex polymerase chain reaction
NPV: Negative predictive value
PJI: Periprosthetic joint infection
PPV: Positive predictive value
